# Photoallylation Mechanism of Dicyanobenzenes with Allyltrimethylsilane in Solution Investigated by Cold UV and IR Spectroscopy in the Gas Phase

**DOI:** 10.1002/asia.202500750

**Published:** 2025-07-31

**Authors:** Ryosuke Goda, Yuma Kitamura, Go Nagamoto, Satoru Muramatsu, Manabu Abe, Yoshiya Inokuchi

**Affiliations:** ^1^ Department of Chemistry Graduate School of Advanced Science and Engineering Hiroshima University Higashi‐Hiroshima 739–8526 Japan

**Keywords:** Cold gas‐phase spectroscopy, Conformation analysis, Dicyanobenzene, Photoallylation, UV photodissociation

## Abstract

The photoallylation of three structural isomers of dicyanobenzene (DCB), 1,4‐DCB, 1,3‐DCB, and 1,2‐DCB, was investigated. Reactivity dependence on the relative position of the cyano groups is acknowledged; 1,3‐DCB exhibits lower reactivity. The protonated reaction intermediates were identified at *m/z* 171 (C_11_H_11_N_2_^+^) for all DCB isomers at comparable intensities. The C_11_H_11_N_2_^+^ intermediates are produced by adding an allyl group to DCB, implying that (1) the reaction is initialized via allyl‐group addition to DCB and the formation of neutral (C_11_H_10_N_2_) intermediates is followed by cyano‐group elimination and (2) the initial allyl‐group addition occurs effectively regardless of the cyano‐group position. UV photodissociation (UVPD) and IR‐UV double resonance (DR) spectra of the C_11_H_11_N_2_^+^ intermediates were measured under cold (∼10 K) gas‐phase conditions and compared with theoretical spectra. The intermediate structures identified from 1,2‐DCB and 1,4‐DCB appear compatible with the subsequent β‐elimination of the cyano group, which is probably the final step of photoallylation. Conversely, the intermediate structure from 1,3‐DCB comprises β hydrogens between the cyano and allyl groups, which can induce the CH/π hydrogen bonds with the cyano and allyl groups. This conformation restricts nucleophilic access to the β hydrogens and suppresses cyano‐group elimination, accounting for the low reactivity of 1,3‐DCB in solution.

## Graphical abstract

The structure of the chemical intermediate in photoallylation of dicyanobenzenes (DCBs) with allyltrimethylsilane (ATMS) in acetonitrile was determined by cold UV and IR spectroscopy in the gas phase. The reaction mechanism was discussed on the basis of the intermediate structure. It was found that the elimination of a CN group from the intermediate is a rate‐limiting step that governs the reactivity of DCBs.

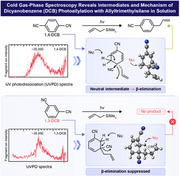

## 1 Introduction

**Scheme 1:**
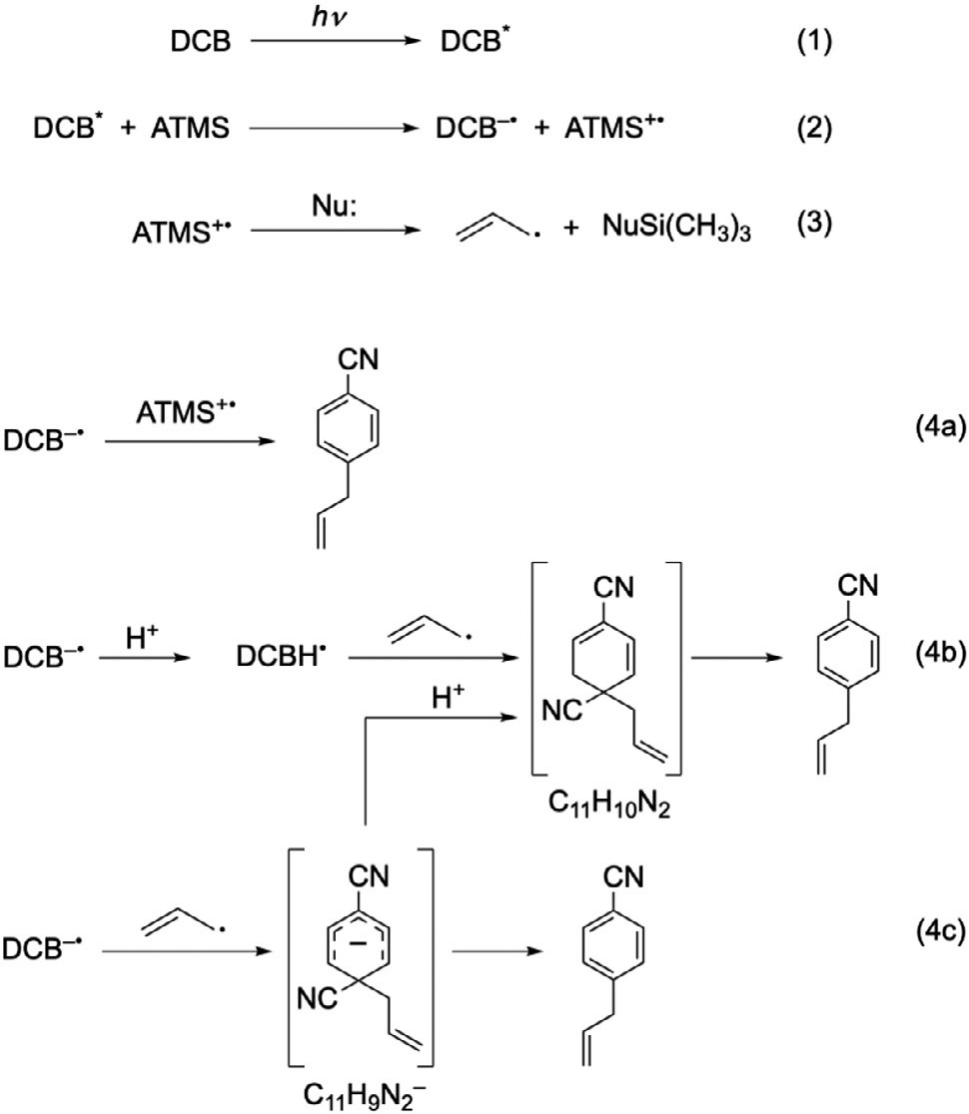
Previously proposed reaction mechanism for photoallylation of DCBs with ATMS.

Over the last few decades, remarkable progress has been made in cold gas‐phase spectroscopy, beginning with the development of cold ion traps.^[^ ^1^, ^2^ ^]^ Cold gas‐phase spectroscopy shows the high resolving power of conformers owing to the narrow bandwidths in electronic transitions and the high structural determination ability of each conformer. Thus, cold gas‐phase spectroscopy has been applied to a variety of complicated ions, including biologically relevant molecules and host‐guest systems.^[^ ^3^, ^4^, ^5^, ^6^, ^7^ ^]^ The usefulness of gas‐phase spectroscopy for the structural determination of ions in the gas‐phase is widely recognized in the physical chemistry community, therefore, the cold gas‐phase spectroscopy of ions can be further generalized to multidisciplinary chemistry. Recently, we focused our attention on gas‐phase spectroscopy of transient species, that is, chemical intermediates in the reactant solution.^[^ ^8^, ^9^ ^]^ For photochemical reactions in solution, transient species formed upon the irradiation have been extensively investigated using time‐resolved spectroscopy.^[^ ^10^ ^]^ Chemical intermediates in solution have also been detected using mass spectrometry (MS), which can determine the molecular weight and atomic composition of the species.^[^ ^11^, ^12^, ^13^, ^14^, ^15^, ^16^, ^17^, ^18^, ^19^, ^20^, ^21^, ^22^, ^23^, ^24^, ^25^, ^26^, ^27^, ^28^, ^29^ ^]^ To determine the electronic and geometric structures, ion‐mobility MS, infrared (IR) photodissociation spectroscopy, and visible/ultraviolet photodissociation spectroscopy were performed on the photochemical intermediates produced in solution and introduced into mass spectrometers.^[^ ^30^, ^31^, ^32^ ^]^ More recently, we studied the chemical intermediates in the photosubstitution of 1,4‐dicyanobenzene (1,4‐DCB) with allyltrimethylsilane (ATMS) in acetonitrile using cold gas‐phase spectroscopy.^[^ ^8^ ^]^ Scheme 1 illustrates the proposed reaction mechanism.^[^ ^33^, ^34^ ^]^ Upon photoirradiation, DCB is promoted to the electronic excited state (Equation 1). Electron transfer occurs between excited DCB (DCB^*^) and ATMS, forming DCB^–•^ and ATMS^+•^ radical ions (Equation 2). The ATMS^+•^ radical cation produces an allyl radical (Equation 3). The DCB^–•^ radical anion reacts with the ATMS^+•^ radical cation or the allyl radical. Finally, one of the two cyano groups is released from the intermediates and the substitution of one cyano group with an allyl group is completed (Equation 4).^[^ ^33^ ^]^ Arakawa et al. conducted an MS study of the chemical intermediates of these photoallylation reactions.^[^ ^34^ ^]^ A chemical intermediate was detected in its protonated form and its ultraviolet photodissociation (UVPD) spectrum was observed; on this basis, its structure was determined.^[^ ^8^ ^]^

**Scheme 2:**
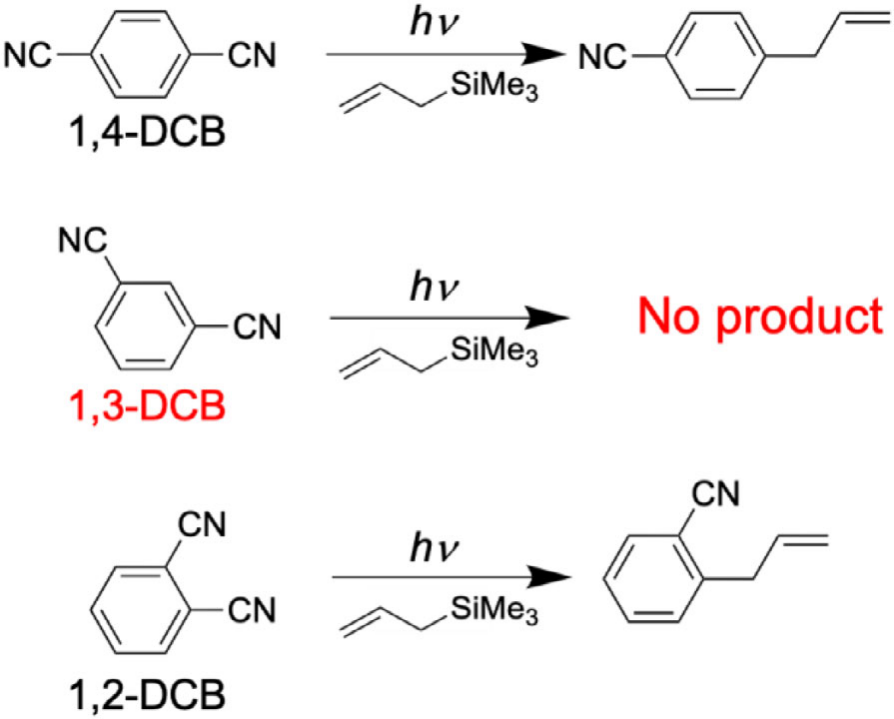
Photoallylation of DCBs with ATMS investigated in this study.

In this study, we extended our gas‐phase investigation of the photochemical intermediates to other structural isomers of DCB, namely, 1,3‐DCB and 1,2‐DCB. Scheme 2 shows the reactants and products involved in the photoallylation of DCBs using the ATMS investigated in this study. A previous study reported that 1,3‐DCB does not react with ATMS.^[^ ^33^ ^]^ The purpose of this study was to detect the chemical intermediates in the photoallylation reaction of DCBs, determine their structure, and elucidate the reaction mechanism and reason for the low reactivity of 1,3‐DCB based on its structure. The mass spectra of the DCBs with ATMS in acetonitrile were measured to detect chemical intermediates. We performed UVPD and IR‐UV double resonance (IR‐UV DR) spectroscopy of the photochemical intermediates to investigate their geometric and electronic structures. These results provide valuable information for a microscopic understanding of photochemical reaction mechanisms.

## 2 Experimental and Computational Methods

The experimental details have been described previously.^[^ ^7^, ^8^ ^]^ Briefly, we used an in‐house electrospray ionization (ESI) source to introduce the reaction intermediates, formed in solution into a vacuum. The ESI source comprised a stainless‐steel needle with an inner diameter of 0.108 mm, fused quartz tube, and 1/16 in. stainless tube. These parts were connected with an epoxy resin. The reactant solution was transferred from a syringe to the ESI source using a 1/16 in. Teflon tube via a syringe pump; the flow rate was 0.5 mL h^−1^. The reactant solution was irradiated onto the quartz section using a Xe lamp (> 250 nm) through an optical fiber and injected into a vacuum chamber. One of the three DCB isomers and ATMS were dissolved in acetonitrile at the concentration of 0.5 × 10^−2^ and 1.5 × 10^−2^ M, respectively. The ions produced by the ESI source were introduced into a cold Paul‐type quadrupole ion trap (QIT) through a vaporization tube and octopole ion guide. The QIT was cooled to ∼4 K using a He cryostat and He buffer gas was continuously introduced into the QIT. The ions were stored in the QIT and cooled translationally and internally by collision with the cold He buffer gas. The vibrational temperature of the trapped ions was estimated to be ∼10 K.^[^ ^35^ ^]^ Ions other than the parent ions of interest could be removed from the QIT by applying an RF potential to the entrance end cap.^[^ ^36^ ^]^ The cold ions in QIT were irradiated using a tunable UV laser (EKSPLA NT342B), and the resulting fragment ions were mass‐analyzed and detected using a homemade time‐of‐flight mass spectrometer.^[^ ^37^ ^]^ The UVPD spectra of the parent ions were obtained by plotting the yields of the fragment ions against the UV laser wavenumber. In the IR‐UV DR experiments, the wavenumber of the UV laser was fixed, and the intensity of the fragment ions was monitored. The output of an IR laser (LaserVision) was introduced to the QIT 100 ns before the UV laser while scanning the wavenumber of the IR laser in the C─H and N─H stretching regions (2700–3500 cm^−1^). The IR‐UV DR spectra were obtained by plotting the ratio of the fragment ion intensity with the IR laser on/off (*I*_on_/*I*_off_) as a function of the wavenumber of the IR laser.^[^ ^7^ ^]^

We also performed density functional theory (DFT) calculations to predict the stable forms and electronic and vibrational transitions of the photochemical intermediates. Initially we carried out initial conformation search for each structural isomer using the CONFLEX high‐performance conformation analysis program^[^ ^38^ ^]^ with the MMFF94s force field to obtain initial structures for DFT calculations. Geometry optimization of stable conformers obtained in the initial conformation search was further performed using the GAUSSIAN 16 program at the M06‐2X/6–311++G(d,p) level.^[^ ^39^ ^]^ Vibrational analysis was performed for the optimized structures at the same calculation level. We conducted time‐dependent DFT (TD‐DFT) calculations at the M06‐2X/6–311++G(d,p) level to determine the electronic transition energy and oscillator strength. The polarizable continuum model (PCM) was used to calculate the species in acetonitrile. The spin densities of the radical species were obtained using Mulliken population analysis.

## 3 Results and Discussion

### 3.1 Formation of Photochemical Intermediates With DCBs and ATMS

**Figure 1:**
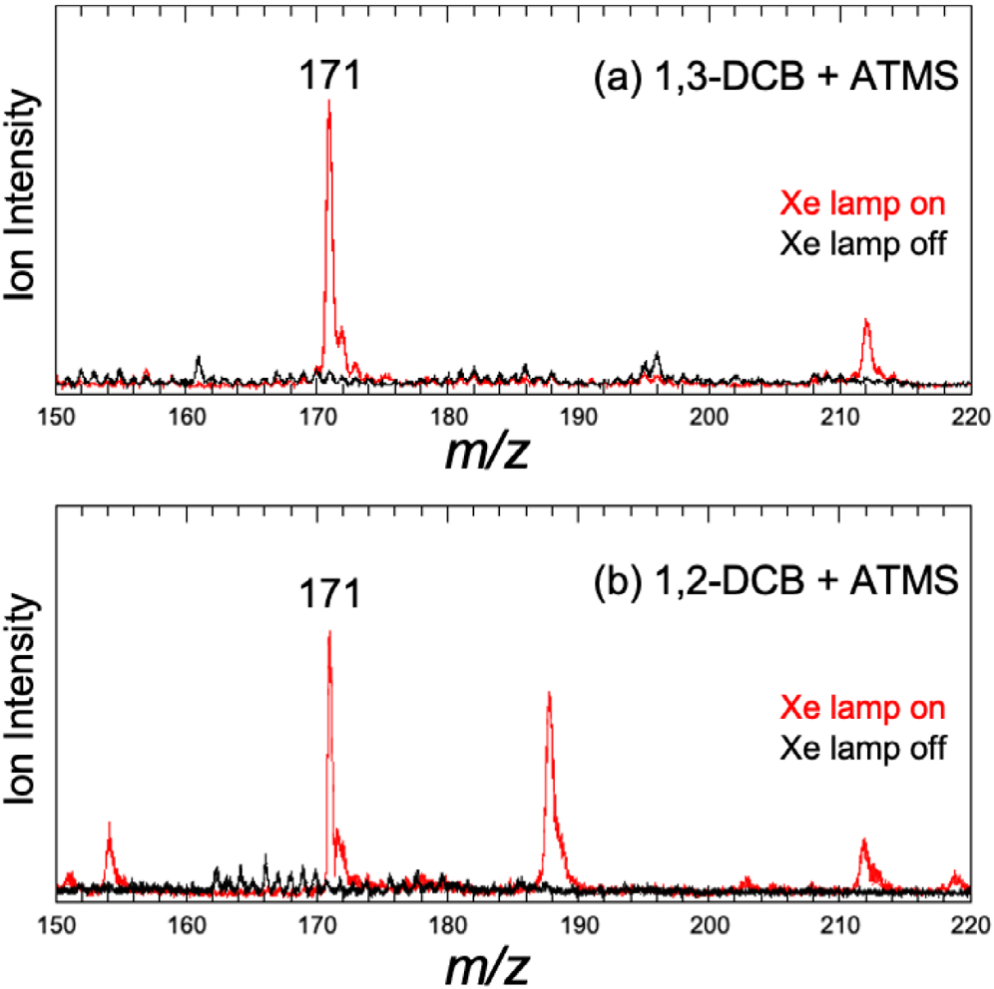
Mass spectra of the a) (1,3‐DCB + ATMS) and b) (1,2‐DCB + ATMS) mixture in acetonitrile, observed with the in‐house mass spectrometer. Red and black curves show the mass spectra with/without irradiation by the Xe lamp, respectively.

First, we examined the formation of photochemical intermediates using DCBs and ATMS by measuring their mass spectra. As reported previously, 1,4‐DCB reacts with ATMS in acetonitrile under Xe lamp irradiation and provides a strong ion signal at *m/z* 171 in the mass spectrum.^[^ ^8^, ^34^ ^]^ Figure S1 shows the absorption spectra of the DCBs in acetonitrile. The maximum absorption was observed at ∼290 nm for 1,4‐DCB and 1,2‐DCB. For 1,3‐DCB, the band maximum was located at ∼288 nm, which was similar to those of 1,4‐DCB and 1,2‐DCB. Figure S2 displays the energy of the (DCB^−•^ and ATMS^+•^) radical ion pairs relative to that of the (DCB and ATMS) neutral pairs in acetonitrile, calculated at the M06‐2X/6–311++G(d,p) level of theory; the radical ion pairs are expected to be formed after UV absorption by DCBs.^[^ ^34^ ^]^ The relative energy of the (DCB^−•^ and ATMS^+•^) radical ion pair was ∼340 kJ mol^−1^ for all DCBs, which is substantially lower than the electronic transition energy of DCBs (∼290 nm, 413 kJ mol^−1^). The results in Figures S1 and S2 suggest that the formation of a radical ion pair is possible for all DCBs. Figure 1 shows the mass spectra of the (DCB and ATMS) mixture in acetonitrile for 1,3‐DCB and 1,2‐DCB measured using our ESI source and mass spectrometer. The red and black curves indicate the mass spectra obtained with and without Xe lamp irradiation, respectively. For both 1,3‐DCB and 1,2‐DCB, a strong signal was observed at *m/z* 171, similar to that observed for 1,4‐DCB.^[^ ^8^ ^]^ The ion at *m/z* 171 can be ascribed to C_11_H_11_N_2_^+^. For 1,4‐DCB, an anionic intermediate (C_11_H_9_N_2_^−^) and/or a neutral intermediate (C_11_H_10_N_2_) was expected to be present during the photoallylation reaction with ATMS.^[^ ^34^ ^]^ The anionic species was not observed in the mass spectra measured in negative mode. However, the intermediate was detected as a protonated form at *m/z* 171 (C_11_H_11_N_2_^+^).^[^ ^8^, ^34^ ^]^ Hence, the ion observed at *m/z* 171 for 1,3‐DCB and 1,2‐DCB (Figure 1) was also related to a chemical intermediate in the photoallylation of DCBs with ATMS.^[^ ^8^, ^34^ ^]^ Although a previous report revealed the much lower reactivity of 1,3‐DCB than that of 1,2‐DCB and 1,4‐DCB,^[^ ^33^ ^]^ our observations indicate that the reaction proceeds, at least up to the formation of the chemical intermediate for 1,3‐DCB. For 1,2‐DCB, a strong signal is observed at *m/z* 188. This ion could be a chemical species related to unidentified products in the previous study.^[^ ^33^ ^]^ To reveal this ion, we should first identify the unidentified product, which is beyond the scope of this study and will be a future task.

### 3.2 Structural Determination of Photochemical Intermediates by Cold Gas‐Phase Spectroscopy

**Figure 2:**
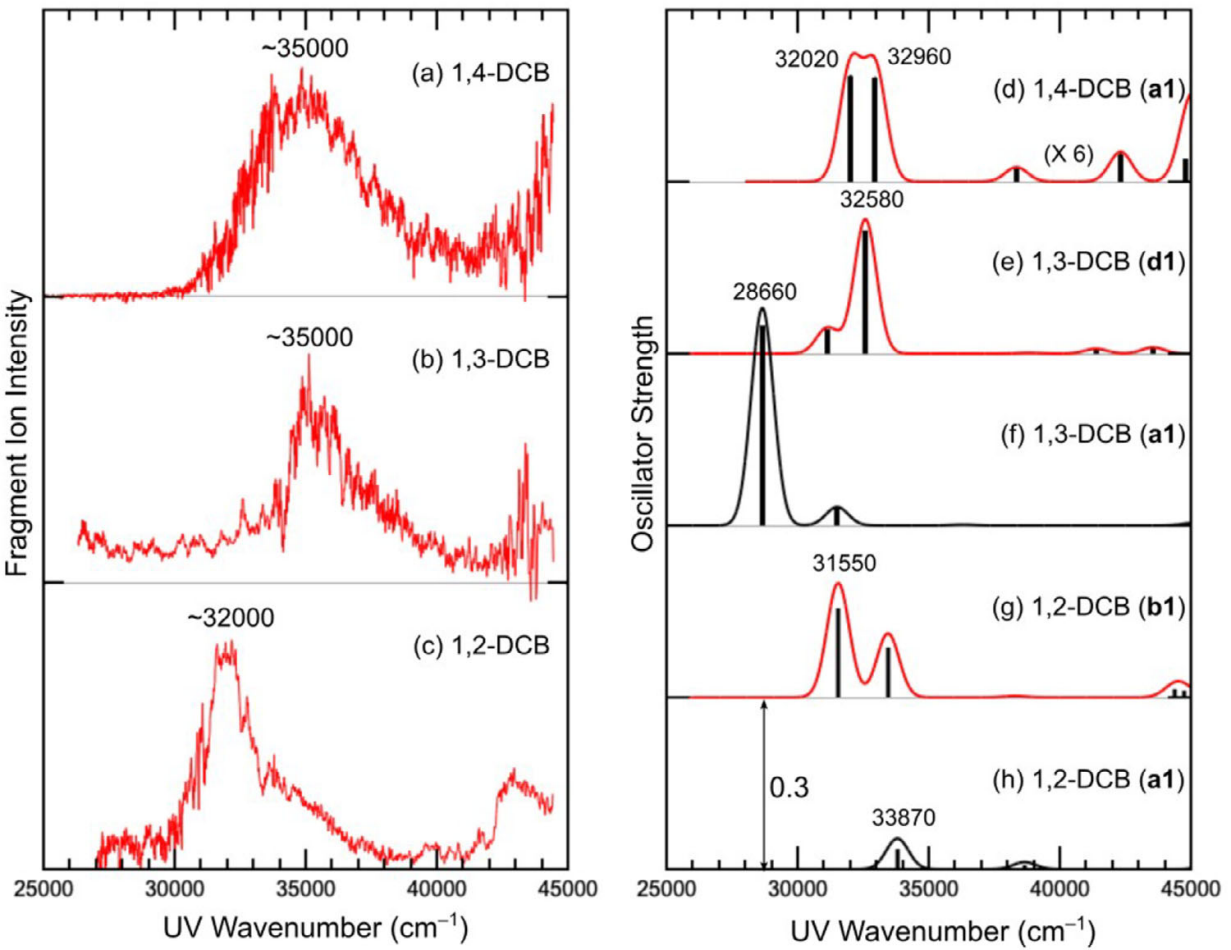
UVPD spectra of the *m/z* 171 ions for a) 1,4‐DCB, b) 1,3‐DCB, and c) 1,2‐DCB. Calculated UV spectra of the stable *m/z* 171 ions for d) 1,4‐DCB, e, f) 1,3‐DCB, and g, h) 1,2‐DCB. The UVPD spectrum of the *m/z* 171 ion for 1,4‐DCB was taken from a previous report (Ref. [8]). The double‐headed arrow in panel h corresponds to the oscillator strength of 0.3. The solid curves in panels d–h were produced by applying Gaussian functions with an FWHM of 1000 cm^−1^ to the calculated electronic transitions.

The structure of the *m/z* 171 ions was determined by UVPD spectroscopy using DFT calculations. The UVPD spectra of the *m/z* 171 ions are shown in Figures 2a–c. The UVPD spectrum of 1,4‐DCB (Figure. 2a) was obtained from a previous report for comparison.^[^ ^8^ ^]^ Because the *m/z* 171 ions were cooled to ∼10 K in the QIT, the broad features of the UVPD spectra could be attributed not to inhomogeneous broadening due to solvent or thermal effects but to the intrinsic nature of the *m/z* 171 ions, such as the lifetime broadening of the electronic excited states, Franck‐Condon envelopes and the presence of multiple excited states/isomers. The *m/z* 171 ion for 1,4‐DCB exhibited a band maximum at ∼35 000 cm^−1^ with an FWHM of ∼5500 cm^−1^ (Figure 2a). The UVPD spectrum of the *m/z* 171 ion for 1,3‐DCB (Figure 2b) showed a band maximum at ∼35 000 cm^−1^ with an FWHM of ∼2900 cm^−1^, which is narrower than that for 1,4‐DCB. For 1,2‐DCB (Figure 2c), the *m/z* 171 ion showed a red shift of the band maximum to ∼32 000 cm^−1^.

**Figure 3:**
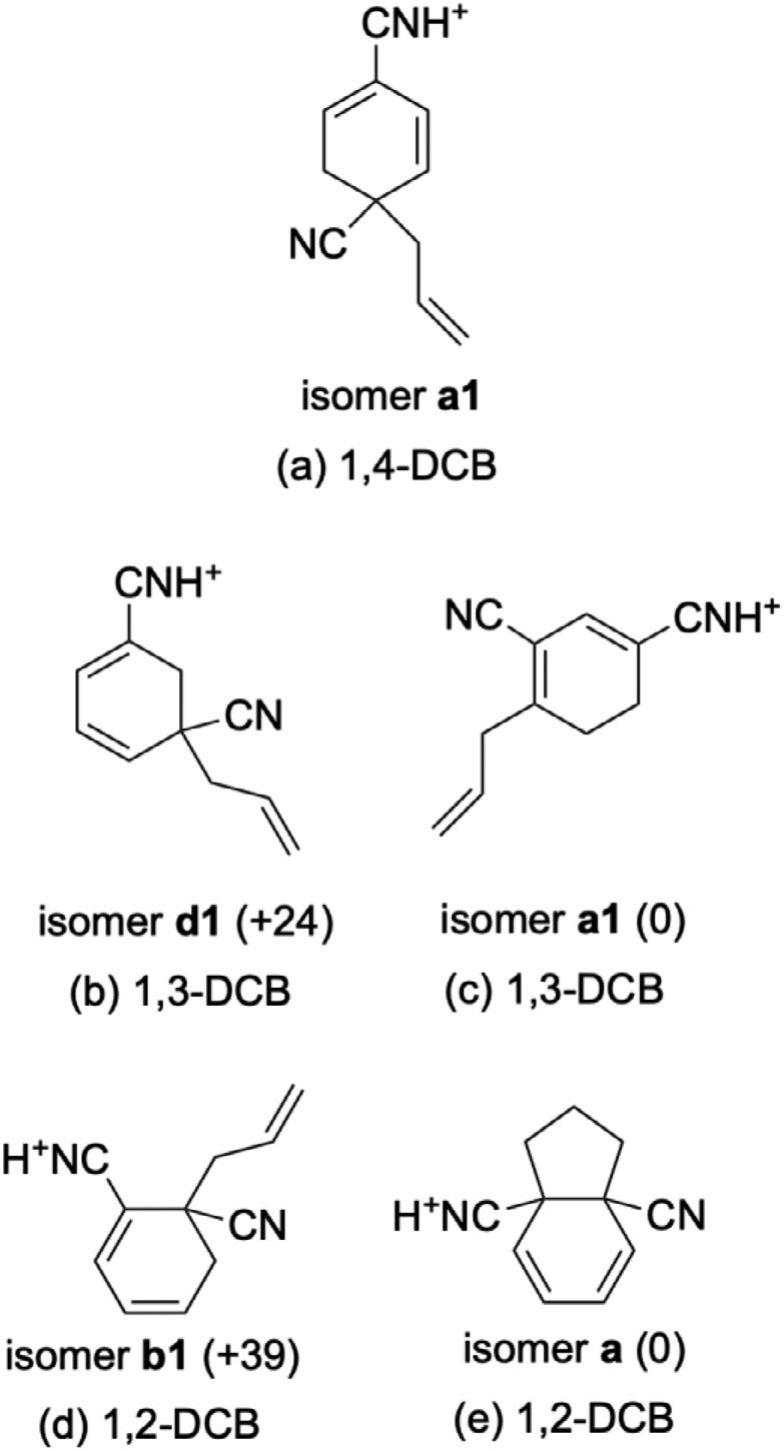
Stable structures of the *m/z* 171 ions for a) 1,4‐DCB, b, c) 1,3‐DCB, and d, e) 1,2‐DCB. The numbers in parentheses in panels b–e indicate the relative total energy of the structural isomers in kJ mol^−1^.

The calculated electronic spectra of the stable isomers of the *m/z* 171 ions are shown in Figures 2d–h. Figure 3 shows the structures of the *m/z* 171 ions optimized in the DFT calculations. The other optimized structures are summarized in Figures S3, S5, and S7 for 1,3‐DCB and 1,2‐DCB; the stable isomers of 1,4‐DCB have been reported previously.^[^ ^8^ ^]^ The *m/z* 171 ions (C_11_H_11_N_2_^+^) were recognized as protonated species of neutral C_11_H_10_N_2_, which could be the chemical intermediate in the photoallylation of DCBs with ATMS formed by the addition of one allyl radical to the DCB^−•^ radical anion.^[^ ^33^, ^34^ ^]^ Previous reports have stated that 1,4‐DCB and 1,2‐DCB provide substitution products (Scheme 2) in high yields (>60 %), whereas 1,3‐DCB is essentially unreactive.^[^ ^33^, ^34^ ^]^ Based on this trend of the reactivity, for 1,4‐DCB and 1,2‐DCB we considered the C_11_H_10_N_2_ chemical intermediates that have the allyl group at the *ipso* position of the CN groups. For the C_11_H_10_N_2_ chemical intermediate of 1,3‐DCB, all the structural isomers with an allyl group bonded directly to the benzene ring were examined by quantum chemical calculations. The alphabetic character of the isomer name of the C_11_H_11_N_2_^+^ ions (**a**, **b**, etc.) indicates the framework of the C_11_H_10_N_2_ portion. The protonation of the C_11_H_10_N_2_ portion occurs in either of the two cyano groups. The numbers in the isomer names (**1** and **2**) indicate the different protonation positions. Hence, isomers **a1** and **a2** have the same C_11_H_10_N_2_ framework with protons at different cyano groups. The most stable isomer in the present DFT calculations was named isomer **a1** for each DCBs.

The UVPD spectra in Figures 2a–c were assigned based on their relative band positions. In a previous study on 1,4‐DCB, the UVPD band at ∼35 000 cm^−1^ was reasonably assigned to isomer **a1** (Figure 3a).^[^ ^8^ ^]^ For 1,3‐DCB, isomer **d1** (Figure 3b) showed strong absorption at almost the same position as that of isomer **a1** of 1,4‐DCB (Figures 2d and e), which reproduced the strong bands in the UVPD spectra of 1,4‐DCB and 1,3‐DCB at the same (∼35 000 cm^−1^) position (Figures 2a and b). Hence, the UVPD spectrum of the *m/z* 171 ion for 1,3‐DCB (Figure 2b) can be ascribed to isomer **d1** in Figure 3b. The broader feature of the UVPD spectrum of 1,4‐DCB (Figure 2a) was attributed to the presence of two comparative electronic transitions predicted to exist between 32 020 and 32 960 cm^−1^ in the TD‐DFT calculations (Figure 2d).

Isomer **d1** of 1,3‐DCB, which has an allyl group at the *ipso* position of the CN group was not the most stable form in the DFT calculations. The most stable structure was isomer **a1** (Figure 3c), in which the allyl group is bonded to the 4‐position of 1,3‐DCB. The calculated UV spectrum of isomer **a1** (Figure 2f) shows a strong transition at 28 660 cm^−1^, which is substantially smaller than those of isomer **a1** of 1,4‐DCB and isomer **d1** of 1,3‐DCB (Figures 2d and e). The result for isomer **a1** for 1,3‐DCB was not consistent with the UVPD trend shown in Figures 2a and b. The structures and calculated electronic spectra of the isomers that were more stable than **d1** are shown in Figure S4. The first to fourth most stable isomers (**a1**, **a2**, **b1**, and **c1**) have an allyl group but not at the *ipso* position of the CN group. Isomer **a2** exhibited a strong transition at a position lower than 33 000 cm^−1^. Isomer **b1** exhibited another strong transition around 30 000 cm^−1^. There was no strong transition for isomer **c1**. The calculated results for isomers **a1**, **a2**, **b1**, and **c1** are not consistent with the UVPD results for 1,3‐DCB (Figure 2b). We also examined the possibility of other isomers of the *m/z* 171 (C_11_H_11_N_2_^+^) ion for 1,3‐DCB with an allyl group at the *ipso* position of the CN groups, such as isomer **d1**. The total energies and electronic transitions of these isomers (isomers **d1**, **d2**, **j1**, **j2**, **l1**, and **l2**) are shown in Figures S5 and S6, respectively. Isomer **d2**, which has the same framework of the C_11_H_10_N_2_ portion as that of **d1**, also shows a strong electronic transition (oscillator strength greater than 0.1) in the 30000–35 000 cm^−1^ region (Figure S6). Hence, isomer **d2** may have contributed to the UVPD spectrum of the *m/z* 171 ion. Conversely, isomers **j1**, **l1**, **j2**, and **l2** in Figure S6 do not exhibit strong absorption in the same region (Figure S6), implying that they are not responsible for the UVPD spectrum of the *m/z* 171 ion of 1,3‐DCB. If the *m/z* 171 ion (C_11_H_11_N_2_^+^) is formed via the protonation of the CN groups of the neutral C_11_H_10_N_2_ intermediate, the structure of the C_11_H_10_N_2_ intermediate for 1,3‐DCB can be reasonably ascribed to the isomer **d** form. The cause of the preferential formation of the less stable **d** form (instead of the **a** form) is discussed in the following section.

For 1,2‐DCB, the most stable isomer, according to the DFT calculations performed in this study (isomer **a1**, Figure 3e), has a five‐membered ring. Isomer **a1** does not have strong absorption at approximately 35000 cm^−1^ (Figure 2h), which is not consistent with the UVPD result of a strong band at ∼32000 cm^−1^ (Figure 2c). The second most stable isomer (isomer **b1**, Figure 3d) was predicted to have a strong band at ∼31550 cm^−1^ (Figure 2g), which is lower in transition energy than that of isomers **a1** and **d1** for 1,4‐DCB and 1,3‐DCB, respectively (Figures 2d and e). The relative band positions of the UVPD spectra (Figures 2a–c) were reproduced from the calculated electronic transitions of isomers **a1** (1,4‐DCB), **d1** (1,3‐DCB), and **b1** (1,2‐DCB) (Figures 2d–g). Hence, the structure of the *m/z* 171 ion for 1,2‐DCB was ascribed to isomer **b1** rather than to isomer **a1**. The total energies and electronic transitions of the other structural isomers for the *m/z* 171 ion of 1,2‐DCB are shown in Figures S7 and S8, respectively. Isomers **b1** and **b2** showed a strong electronic transition (oscillator strength greater than 0.1) in the 30000–35000 cm^−1^ region. Isomer **b2** might also be responsible for the UVPD spectrum of 1,2‐DCB. Hence, the structure of the neutral C_11_H_10_N_2_ intermediate for 1,2‐DCB can reasonably be ascribed to isomer **b**.

**Figure 4:**
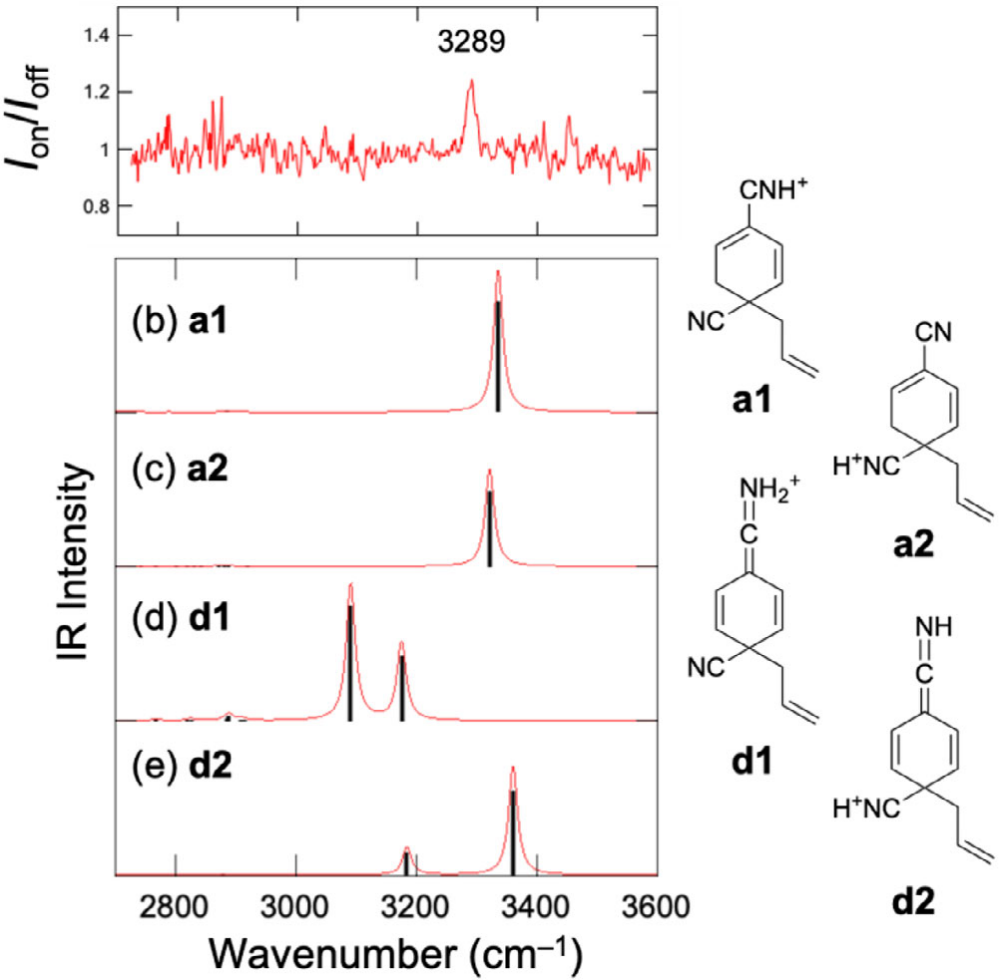
a) IR‐UV DR spectrum of the *m/z* 171 ion for 1,4‐DCB. b–e) Calculated IR spectra of the stable structures. The red curves in panels b–e were reproduced by applying Lorentzian functions with an FWHM of 20 cm^−1^ to the calculated IR transitions.

Previously, two structural isomers were proposed to exist as neutral intermediates for 1,4‐DCB (isomers **4** and **4′** in Scheme 2 of Ref. [34]).^[^ ^34^ ^]^ The protonated forms of isomer **4** (**b1**, **b2**, **c1**, and **c2** in Figure S2 of Ref. [8]) did not exhibit strong absorption at approximately 35 000 cm^−1^ (Figure S4 of Ref. [8]), therefore, we excluded the possibility of isomer **4** for the structure of the intermediate structure.^[^ ^8^ ^]^ Conversely, isomer **4′** was predicted to have an absorption of approximately 35000 cm^−1^ when protonated.^[^ ^34^ ^]^ This is also consistent with the UVPD results for 1,4‐DCB (Figure 2a). We attempted to confirm the structural assignment of the *m/z* 171 ion for 1,4‐DCB using IR‐UV DR spectroscopy. Figure 4a shows the IR‐UV DR spectrum of the *m/z* 171 ion for 1,4‐DCB. The wavenumber of the UV laser was fixed at 35336 cm^−1^ (283.00 nm). In the spectrum, a gain signal was observed at 3289 cm^−1^. The gain signal is ascribed to the UV excitation of hot ions formed via intramolecular vibrational energy redistribution (IVR) following the IR excitation of cold ions.^[^ ^5^ ^]^ Figures 4b–e display the calculated IR spectra of the stable isomers of the *m/z* 171 ion. Previously, we ascribed the UVPD spectrum to isomers **a1** and/or **a2** based on the UV band position.^[^ ^8^ ^]^ The calculated IR spectra of isomers **a1** and **a2** showed a strong band in the 3200–3400 cm^−1^ region (Figures 4b and c). This band was assigned to the N–H stretching vibration of the protonated CN group (–CNH^+^) present in these isomers. The IR spectra of isomers **a1** and **a2** are consistent with the IR‐UV result (Figures 4b and c). Isomers **d1** and **d2** in Figure 4 are the protonated forms of isomer **4′** proposed in the previous study.^[^ ^34^ ^]^ In isomers **d1** and **d2**, there are two N–H bonds. The calculated IR spectra of isomers **d1** and **d2** show two N–H stretching vibrations, as shown in Figures 4d and e. These calculation results of isomers **d1** and **d2** are not consistent with the IR‐UV result. The results in Figure 4 indicate that the assignment of the structure of the *m/z* 171 ion to isomers **a1** and/or **a2** is reasonable, and that isomer **d** can be excluded from the structure of the chemical intermediate for 1,4‐DCB.

### 3.3 Plausible Reaction Mechanism for Photoallylation of DCBs With ATMS

**Figure 5:**
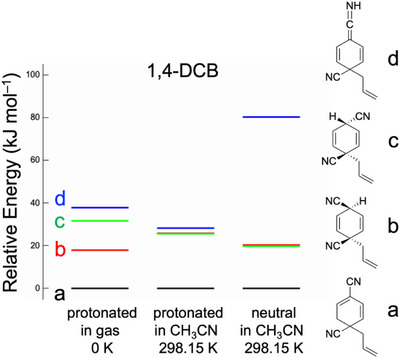
Relative energy of the photochemical intermediates for 1,4‐DCB for three systems: protonated form (C_11_H_11_N_2_^+^) in the gas‐phase at 0 K and in acetonitrile at 298.15 K, and neutral form (C_11_H_10_N_2_) in acetonitrile at 298.15 K. The calculations were performed at the M06‐2X/6–311++G(d,p) level of theory. PCM was used for the calculations in acetonitrile. The Gibbs energy was used to estimate the relative energy among the stable isomers at 298.15 K.

As mentioned above, we considered that the neutral intermediates (C_11_H_10_N_2_) in solution would be detected in their protonated forms (C_11_H_11_N_2_^+^) in our gas‐phase measurements. The relationship of the stability between the structures of the neutral intermediates present in the reactant solution and those of their protonated forms determined by gas‐phase spectroscopy must be clarified. We performed quantum chemical calculations on the neutral and protonated forms in the gas‐phase and acetonitrile solution. Figure 5 shows the relative energies of the structural isomers of the protonated forms (C_11_H_11_N_2_^+^) in the gas‐phase at 0 K and in acetonitrile at 298.15 K and the neutral forms (C_11_H_10_N_2_) in acetonitrile at 298.15 K for 1,4‐DCB. For all three systems, isomer **a** was the most stable among the four structural isomers (isomers **a**–**d**). The UVPD and IR‐UV DR results in Figures 2 and 4 indicate that isomer **a1** and/or **a2** are the structure of the protonated species. Hence, we concluded that the neutral C_11_H_10_N_2_ intermediate in the photoallylation of 1,4‐DCB with ATMS had an isomeric **a** form.

In a previous study, Nakanishi et al. suggested three possibilities for coupling of DCB^−•^ with ATMS^+•^ (Scheme 1, Equation 4a–c): (a) direct coupling between DCB^−•^ and ATMS^+•^, (b) coupling between protonated DCB^−•^ (DCBH^•^) and the allyl radical produced from ATMS^+•^, and (c) coupling between DCB^−•^ and the allyl radical.^[^ ^33^ ^]^ The structural analysis of the substitution products confirmed that the allyl radical was involved in the reaction, thereby excluding the first possibility (Equation 4a in Scheme 1). The second and third possibilities (Equations 4b and c in Scheme 1) proceed via the formation of neutral (C_11_H_10_N_2_) and anionic (C_11_H_9_N_2_^−^) intermediates, respectively. In our previous study, no anionic species were observed in MS measurements of the reactant solution.^[^ ^8^ ^]^ This suggests that the lifetimes of the DCB^−•^ radical anion and anionic (C_11_H_9_N_2_^−^) intermediate (if any) in acetonitrile were too short to be detected by MS. It is not possible to determine which of the second and third possibilities is more plausible based on gas‐phase results. However, we propose that the photoallylation reaction proceeds via a neutral (C_11_H_10_N_2_) intermediate, which has a longer lifetime than that of the anionic transient species in acetonitrile. Considering the sampling time of the irradiated reactant solution into vacuum in our mass spectrometer (∼77 s),^[^ ^8^ ^]^ the lifetime of the neutral (C_11_H_10_N_2_) intermediate in acetonitrile should be substantially longer than ∼77 s. The elimination of the cyano group from the neutral (C_11_H_10_N_2_) intermediate is the rate‐limiting step in the photoallylation of DCB.

**Figure 6:**
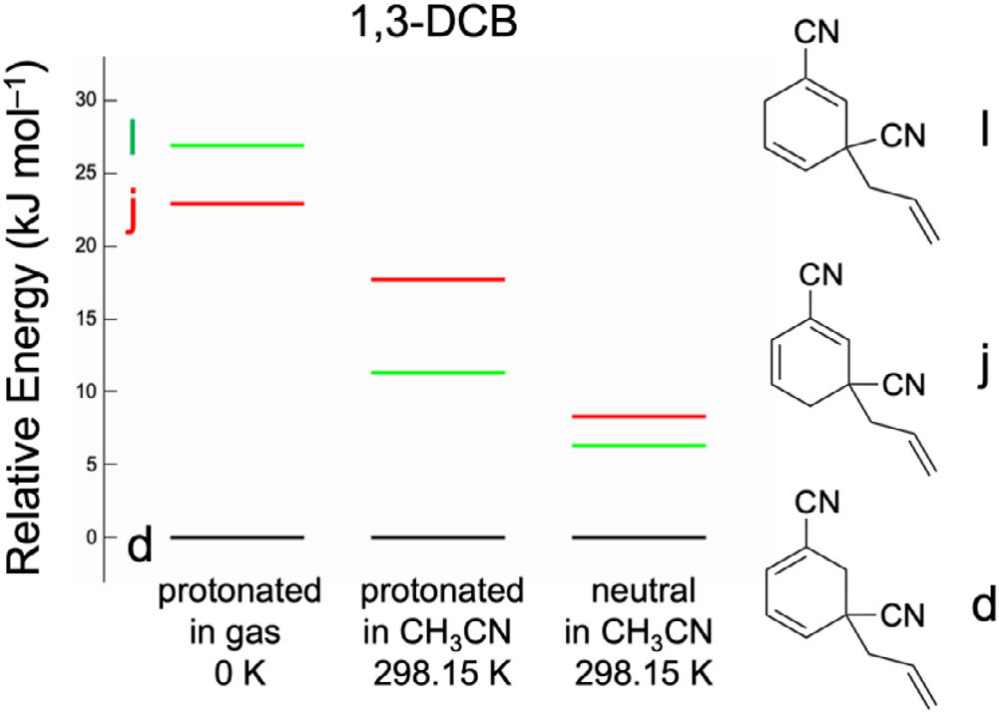
Relative energy of the photochemical intermediates for 1,3‐DCB for three systems: protonated form (C_11_H_11_N_2_^+^) in the gas‐phase at 0 K and in acetonitrile at 298.15 K, and neutral form (C_11_H_10_N_2_) in acetonitrile at 298.15 K. The calculations were performed at the M06‐2X/6–311++G(d,p) level of theory. PCM was used for the calculations in acetonitrile. The Gibbs energy was used to estimate the relative energy among the stable isomers at 298.15 K.

**Figure 7:**
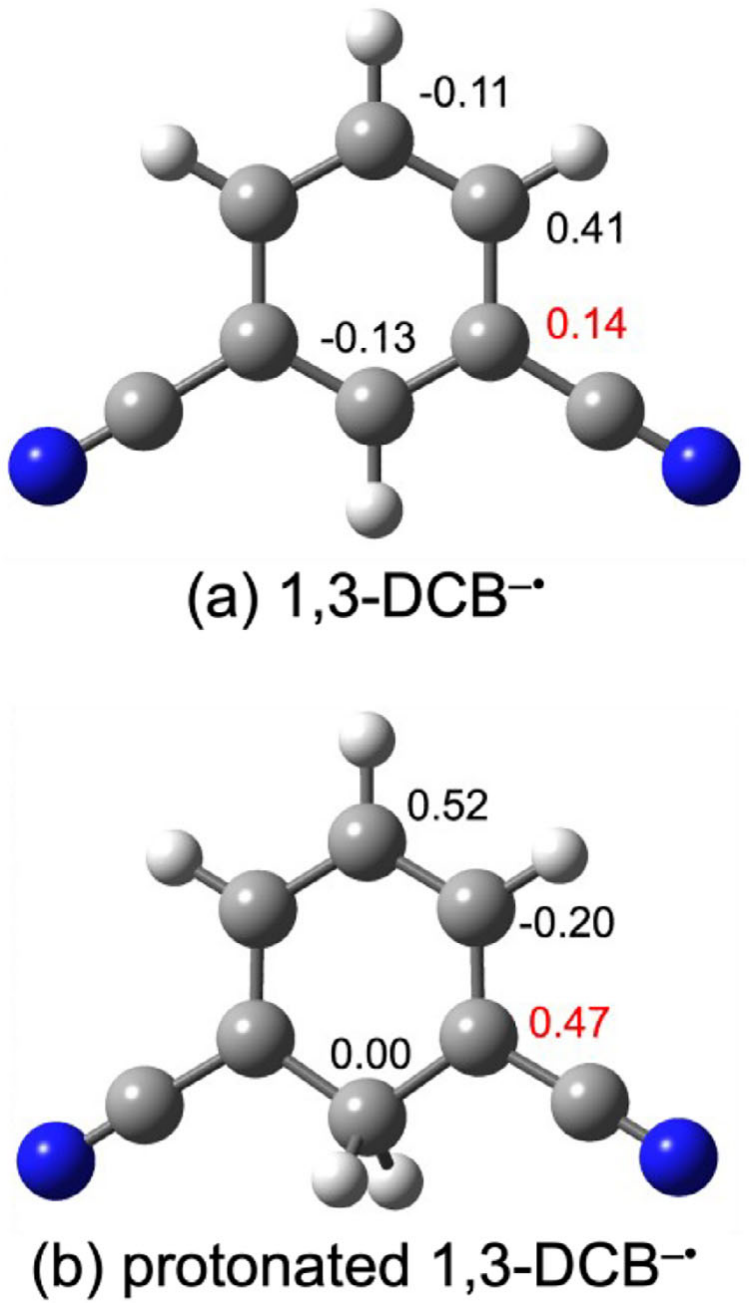
Spin density of the a) 1,3‐DCB^–•^ radical anion and b) 1,3‐DCBH^•^ radical in acetonitrile. The calculations were performed at the M06‐2X/6–311++G d,p) level of theory. PCM was used for the calculations in acetonitrile. The spin density with hydrogen atoms is summed into neighboring carbon atoms. Color code: gray (carbon), blue (nitrogen), and white (hydrogen).

Figure 6 shows the relative energies and structures of the stable isomers of 1,3‐DCB under the constraint that the allyl group is at the *ipso* position of the cyano groups. Isomer **d** was the most stable of all systems. The UVPD spectrum of the protonated form was assigned to isomer **d1** (Figure 2). Hence, it is probable that isomer **d** of C_11_H_10_N_2_ exists as a neutral intermediate in the photoallylation of 1,3‐DCB. Notably, a previous study emphasized that the 1,3‐DCB radical anion (1,3‐DCB^−•^) has a high spin density at the 4‐position.^[^ ^33^ ^]^ This implied the effective formation of isomer **a** of C_11_H_10_N_2_ and isomer **a1** of C_11_H_11_N_2_^+^, in which the allyl group was bonded to the 4‐position of 1,3‐DCB. However, the UVPD results suggested that isomer **d1** was present instead of **a1**. This was ascribed to the reaction mechanism following the formation of the 1,3‐DCB^−•^ radical anion. As shown in Scheme 1, the allyl radical reacts with the 1,3‐DCB^−•^ radical anion or the protonated 1,3‐DCB^−•^ radical (1,3‐DCBH^•^). We examined theoretically the spin density of 1,3‐DCB^−•^ and 1,3‐DCBH^•^ (Figure 7). For 1,3‐DCBH^•^, the isomer in which the proton was bonded to the 2‐position was calculated as the representative isomer (Figure 7b); this structural isomer was most likely converted to isomer **d** (rather than isomer **j** or **l**). The 1,3‐DCB^−•^ radical anion has the highest spin density (0.41) at the 4‐ (and 6‐) position (Figure 7a). Conversely, the spin density of the 1,3‐DCBH^•^ radical was higher (0.47) at the 1‐ (and 3‐) positions than that at the 4‐position (Figure 7b). Hence, it is probable that the 1,3‐DCB^−•^ radical anion was spontaneously protonated and the allyl radical was added to the 1,3‐DCBH^•^ radical (Equation 4b in Scheme 1), leading to the formation of isomer **d**. Table 1 lists the calculated stabilization energies upon protonation (proton affinity) of the DCB^−•^ radical anion. The 1,3‐DCB^−•^ radical anion has the highest value among the three DCB^−•^ radical anions. This partially supports the prompt protonation of the 1,3‐DCB^−•^ radical anions.

**Table 1:** Calculated stabilization energy (*ΔG*) of the DCB^–•^ radical anion upon protonation at 298.15 K in acetonitrile.

	*ΔG* ^a^ ^)^ (kJ mol^−1^)
1,4‐DCB^−•^	1143
1,3‐DCB^−•^	1168
1,2‐DCB^−•^	1140

^a)^ The calculations were performed at the M06‐2X/6–311++G(d,p) level of theory. PCM was used for the calculations in acetonitrile.

**Figure 8:**
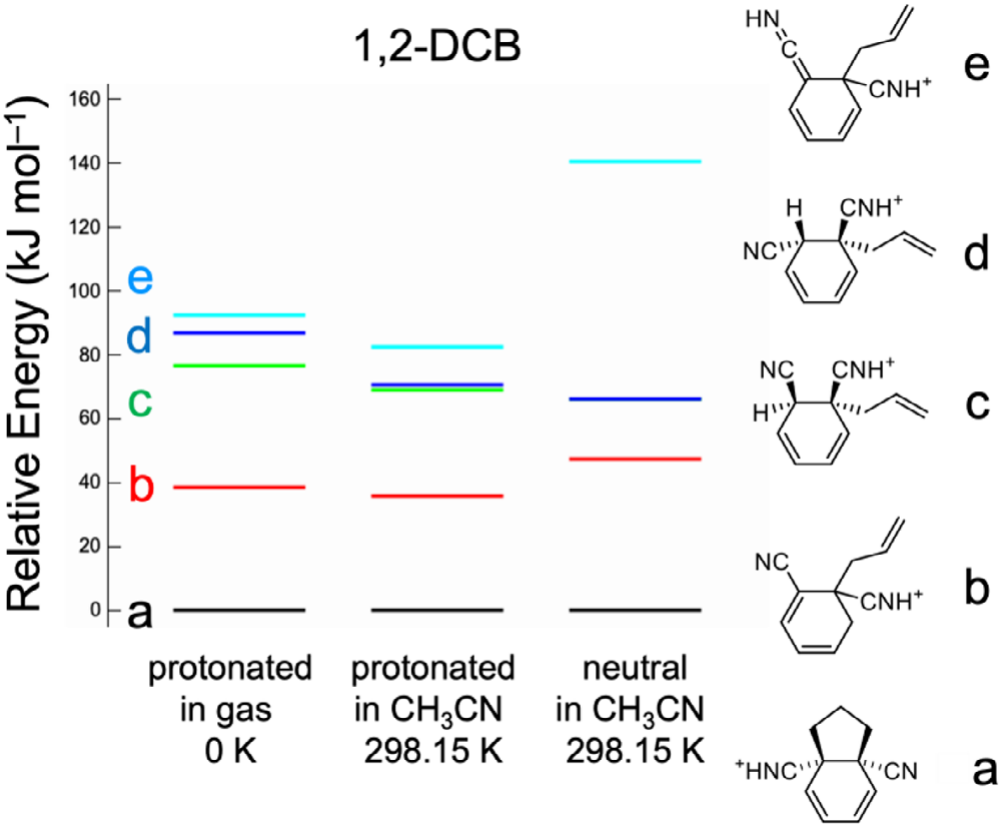
Relative energy of the photochemical intermediates for 1,2‐DCB for three systems: protonated form (C_11_H_11_N_2_^+^) in the gas‐phase at 0 K and in acetonitrile at 298.15 K, and neutral form (C_11_H_10_N_2_) in acetonitrile at 298.15 K. The calculations were performed at the M06‐2X/6–311++G d,p) level of theory. PCM was used for the calculations in acetonitrile. The Gibbs energy was used to estimate the relative energy among the stable isomers at 298.15 K.

**Scheme 3:**
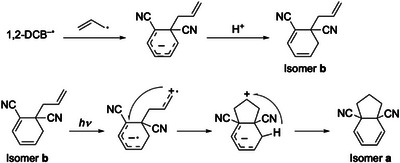
Plausible mechanism for the formation of the *m/z* 171 ion for 1,2‐DCB.

The relative energies and structures of the stable isomers of the 1,2‐DCB intermediates are shown in Figure 8. Isomer **a** was the most stable form for all three systems. However, the UVPD results suggested the presence of isomer **b1** in the gas‐phase. Isomer **b** was the second most stable form in the three systems. Hence, the detection of isomer **b1** by gas‐phase spectroscopy was not driven by its stability in acetonitrile or in the gas‐phase. We attributed the formation of isomer **b** rather than isomer **a** to the formation process. Scheme 3 shows a plausible mechanism for the formation of isomer **b** and **a** of 1,2‐DCB. The 1,2‐DCB^−•^ radical anion (or protonated 1,2‐DCB^−•^) reacts with the allyl radical; in this process, isomer **b** is formed instead of the cyclic isomer **a**, based on the analogy between 1,4‐DCB and 1,3‐DCB. For isomer **a** to form isomer **b**, absorption of the second photon by isomer **b** is required, followed by intramolecular electron transfer and cyclization. In addition, the intramolecular migration of hydrogen atoms should occur to produce isomer **a**. The last process is unlikely to occur efficiently under the current experimental conditions, thus producing isomer **b** as the dominant intermediate.

**Figure 9:**
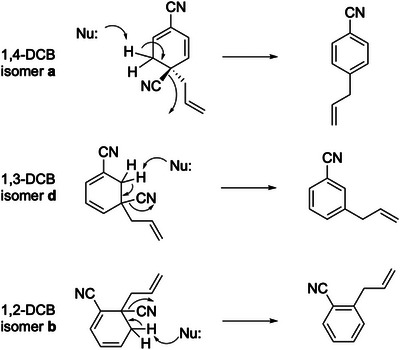
β‐Elimination of the cyano group from the neutral C_11_H_10_N_2_ intermediates in the photoallylation of DCBs.

Finally, we discuss the reaction mechanism involved in the photoallylation of DCBs with ATMS and a plausible reason for the low reactivity of 1,3‐DCB. To complete the photoallylation of DCBs, the cyano group bonded to the *ipso* position of the allyl group should be eliminated from the neutral intermediates. Figure 9 shows the structures of the neutral intermediates determined by gas‐phase spectroscopy of the protonated forms. The structural isomers shown in Figure 9 appear suitable for β‐elimination. A nucleophile (Nu:) attacks the hydrogen atoms at the β position, and the cyano group is eliminated. The photoallylation reaction is finalized by the β‐elimination mechanism following the formation of C_11_H_10_N_2_ intermediates.

**Figure 10:**
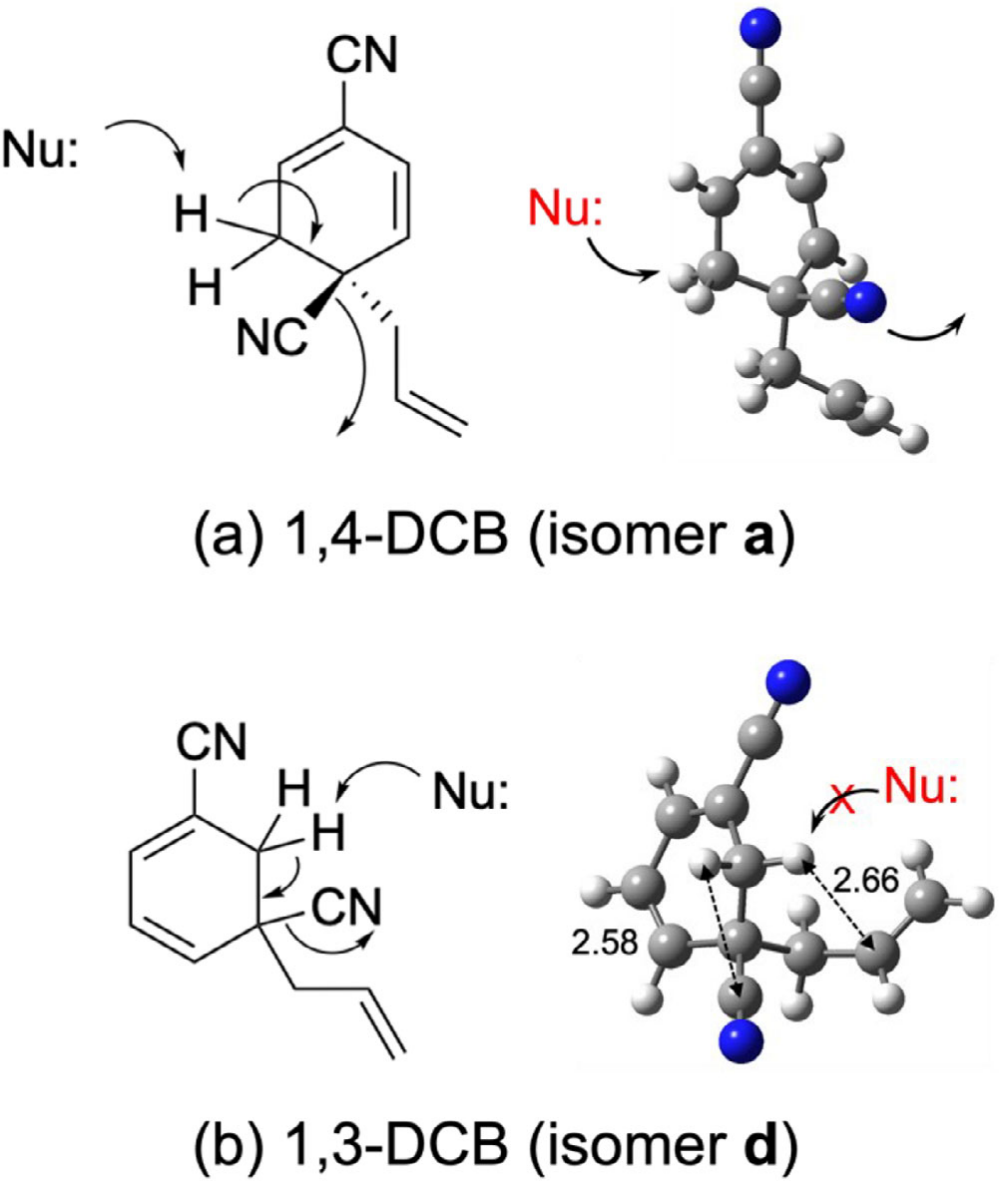
Conformation of the neutral intermediates in acetonitrile at 298.15 K for a) 1,4‐DCB and b) 1,3‐DCB. The numbers in panel b indicate the distance (Å) between the H and C atoms. Color code: gray (carbon), blue (nitrogen), and white (hydrogen). Geometry optimization and vibrational analysis were performed at the M06‐2X/6–311++G(d,p) level of theory with the PCM (acetonitrile).

Figure 10 shows the conformations of the chemical intermediates of 1,4‐DCB and 1,3‐DCB (isomers **a** and **d**, respectively). For 1,4‐DCB (Figure 10a), the β hydrogen atoms are located on one side of the six‐membered ring, opposite to the leaving cyano group. In this conformation, a nucleophile (Nu:) can easily access the β hydrogen atoms. In isomer **d** of 1,3‐DCB (Figure 10b), the β hydrogen atoms are located at a relatively crowded position between the allyl and cyano groups. In particular, the distance between a hydrogen atom at the β position and the carbon atom of the allyl group is 2.66 Å (see Figure 10b), suggesting the formation of a CH/π bond.^[^ ^40^ ^]^ Another hydrogen atom at the β position has a closer distance with the cyano group (2.58 Å, Figure 10b). The conformation of isomer **d** of 1,3‐DCB prevents a Nu: from coming close to the β hydrogen atoms owing to steric hindrance from the allyl and cyano groups. This could suppress the β‐elimination of the cyano group and the substitution reaction for 1,3‐DCB.

**Scheme 4:**
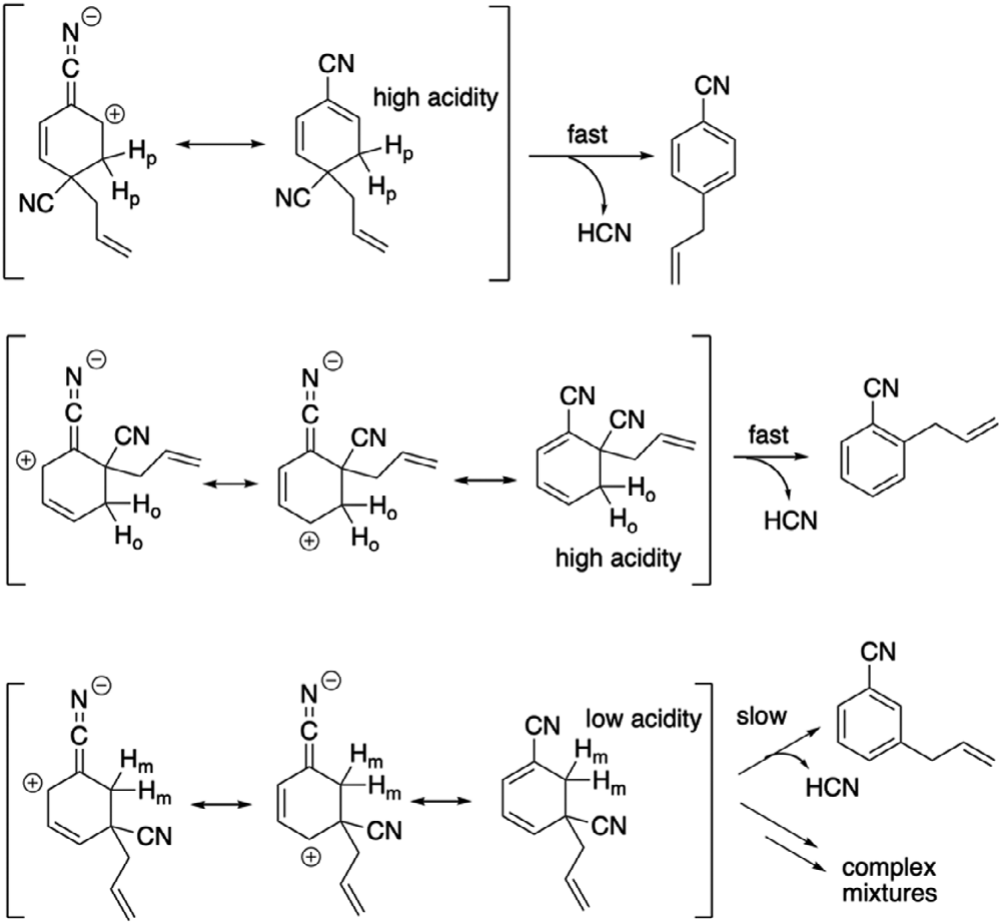
Resonance structures of the C_11_H_10_N_2_ intermediates.

Difference in the reactivity between the DCB structural isomers can also be attributed to the acidity of departing β hydrogen atoms in their C_11_H_10_N_2_ intermediates. In the reactions of 1,4‐DCB and 1,2‐DCB with ATMS, the intermediates formed have positively charged carbons adjacent to the carbon atoms bearing the leaving hydrogens (H_o_, H_p_), as illustrated by their respective resonance structures (Scheme 4). This results in higher acidity of H_o_ and H_p_, promoting rapid elimination of HCN and formation of stable substitution products driven by the aromaticity of the benzene ring. In contrast, in the case of the intermediate derived from 1,3‐DCB, the carbon adjacent to the carbon bearing H_m_ does not develop a positive charge. Consequently, H_m_ is less acidic compared to H_o_ and H_p_, leading to a slower HCN elimination reaction. This delay likely allows side reactions to occur, resulting in complex product mixtures. Indeed, when the photoreaction of 1,3‐DCB with ATMS was conducted in deuterated acetonitrile, consumption of 1,3‐DCB was observed, but no adduct product with ATMS was detected. Instead, broad NMR signals derived from complex products were observed (Figure S9).

## 4 Conclusion

In this study, we demonstrated that cold gas‐phase spectroscopy can be used to examine the reaction mechanism in solution. We detected chemical intermediates in the photoallylation reaction of DCBs with ATMS in acetonitrile as protonated forms in the mass spectra. We measured the UVPD spectra of the protonated intermediates at *m/z* 171 and determined their structures by comparing the UVPD spectra with theoretical spectra. We then derived the structures of the neutral C_11_H_10_N_2_ intermediates formed in acetonitrile based on the structures of the protonated species. The photoallylation reaction proceeded via the formation of long‐lived neutral C_11_H_10_N_2_ intermediates and was finalized by the β‐elimination of the cyano group from the C_11_H_10_N_2_ intermediates. In the conformation of the intermediate for 1,3‐DCB, however, the β hydrogen atoms are located between the cyano and allyl groups and can have CH/π hydrogen bonds with the cyano and allyl groups. This conformation effectively suppresses the access of a nucleophile to the β hydrogen atoms and β‐elimination of the cyano group, which results in low reactivity for 1,3‐DCB. Along with conventional analysis for solutions,^[^ ^41^ ^]^ gas‐phase spectroscopy is expected to continue to be used as a new method for clarifying the mechanism of chemical reactions in solution.

## Supporting Information

Absorption spectra of the DCB structural isomers in acetonitrile. Calculated energies of the (DCB and ATMS) and (DCB^−•^ and ATMS^+•^) pairs in acetonitrile. The structures and electronic transitions of the stable isomers of the cationic (C_11_H_11_N_2_^+^) intermediates of 1,3‐DCB and 1,2‐DCB obtained by DFT calculations. ^1^H NMR spectra of 1,3‐DCB, 1,4‐DCB, and 1,2‐DCB with ATMS in CD_3_CN.

## Conflict of Interests

The authors declare no conflict of interest.

## Supporting information



Supporting Information

## Data Availability

The data that support the findings of this study are available from the corresponding author upon reasonable request.
