# Supplementary material for: Photoallylation Mechanism of Dicyanobenzenes with Allyltrimethylsilane in Solution Investigated by Cold UV and IR Spectroscopy in the Gas Phase
Source: Chem Asian J. 2025 Jul 31;20(18):e00750. doi: 10.1002/asia.202500750 (PMC12450035; doi:10.1002/asia.202500750)
Supplement: Supplementary file 1 — Supporting Information [file ASIA-20-e00750-s001.pdf]

Photoallylation Mechanism of Dicyanobenzenes with Allyltrimethylsilane in Solution Investigated by Cold UV and IR Spectroscopy in the Gas Phase

Ryosuke Goda, Yuma Kitamura, Go Nagamoto, Satoru Muramatsu, Manabu Abe, and Yoshiya Inokuchi*

Department of Chemistry, Graduate School of Advanced Science and Engineering, Hiroshima University, Higashi-Hiroshima 739-8526, Japan.

Supporting Information

*Corresponding author. E-mail: y-inokuchi@hiroshima-u.ac.jp

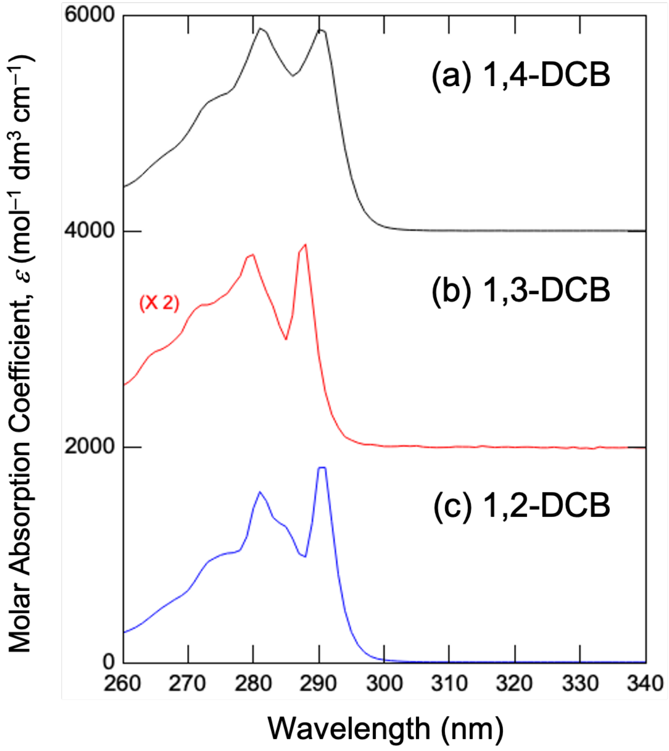

Figure S1. Absorption spectra of the DCN structural isomers in acetonitrile: (a) 1,4-DCB, (b) 1,3-DCB, and (c) 1,2-DCB.

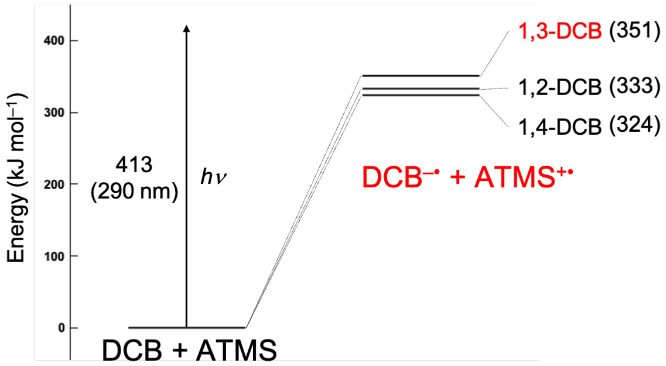

Figure S2. Energetics of (DCN + ATMS) and ($DCN^{-\bullet}$ + $ATMS^{+\bullet}$) in acetonitrile calculated at the M06-2X/6-311++G(d,p) level of theory. The total energy of each species was corrected with the zero-point vibrational energy. PCM was used for the energy in acetonitrile solution.

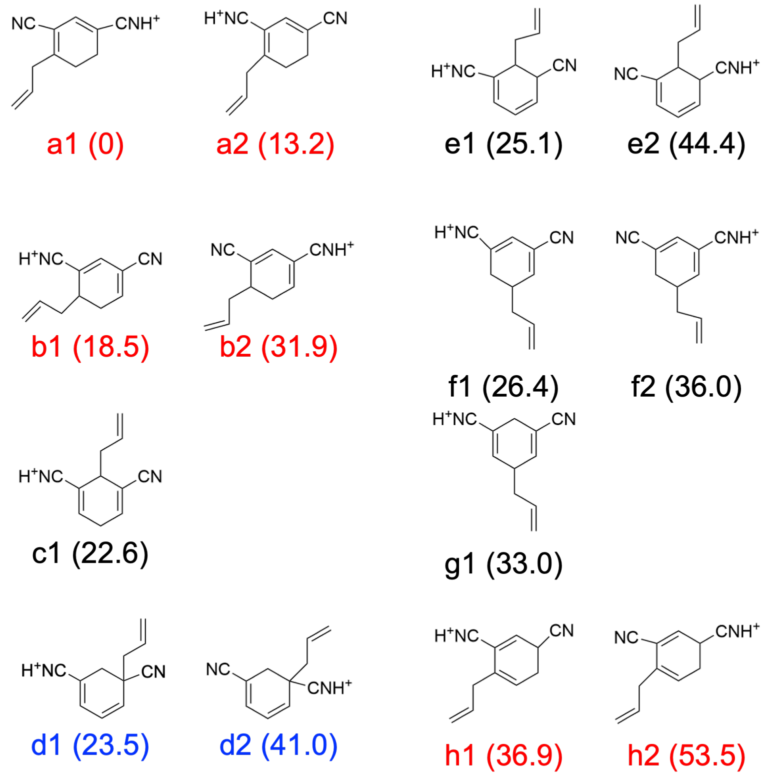

Figure S3. The stable isomers of the *m/z* 171 ion for 1,3-DCB. The calculations were done at the M06-2X/6-311++G(d,p) level. The numbers in parentheses indicate the total energy (kJ mol^{-1}) of the isomers relative to that of isomer **a1**. The total energy was corrected with the zero-point vibrational energy.

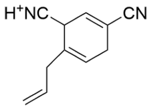

i1 (45.7)

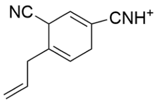

i2 (45.9)

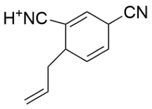

m1 (52.2)

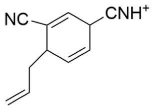

m2 (58.1)

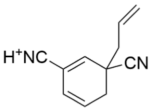

j1 (46.4)

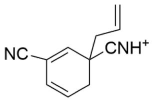

j2 (51.1)

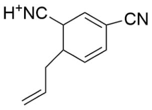

k1 (56.9)

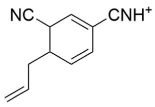

k2 (47.9)

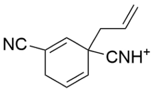

l1 (50.4)

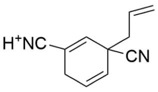

l2 (51.1)

Figure S3. *Cont.*

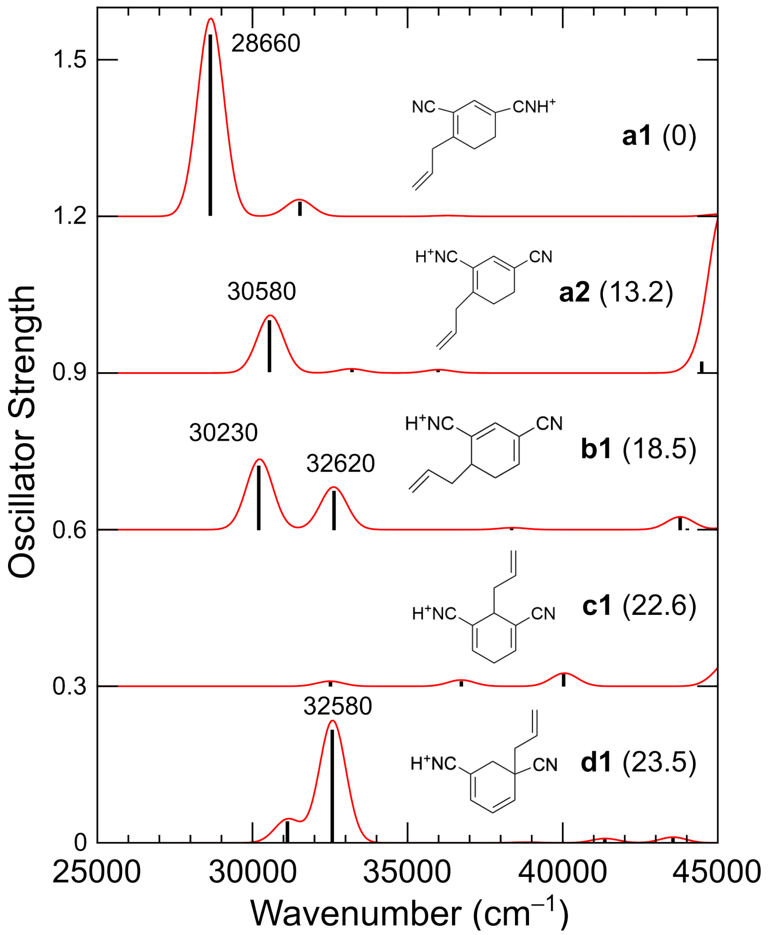

Figure S4. The electronic spectra calculated for the most stable five isomers of the *m/z* 171 ion for 1,3-DCB. The calculations were done at the M06-2X/6-311++G(d,p) level. The numbers in parentheses indicate the total energy (kJ mol^{-1}) of the isomers relative to that of isomer **a1**. The total energy was corrected with the zero-point vibrational energy.

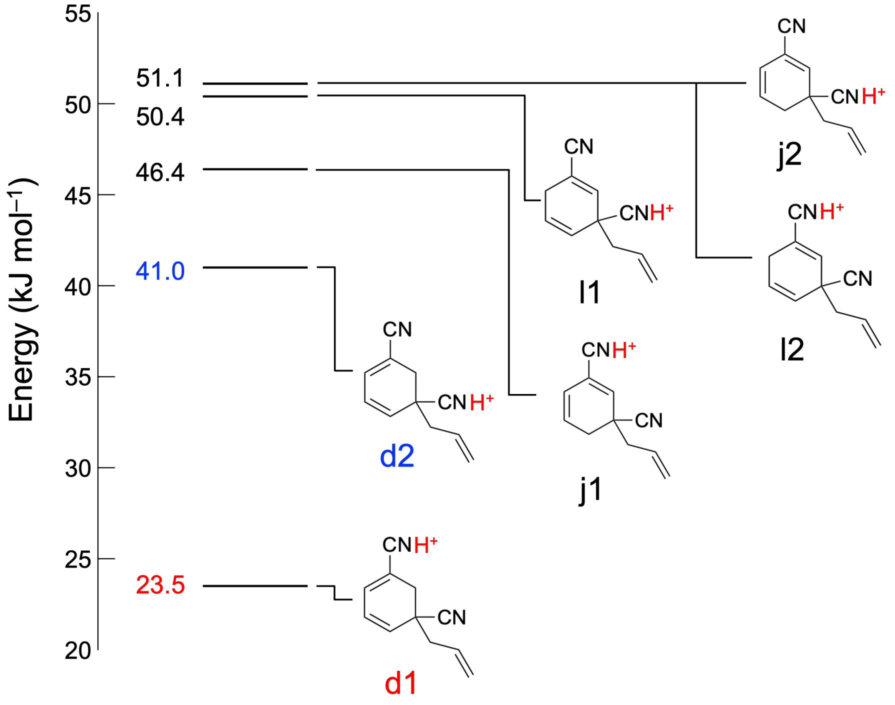

Figure S5. The structure and energy of the stable isomers of the *m/z* 171 ions with the allyl group at the *ipso* position of the cyano group for 1,3-DCB. The calculations were done at the M06-2X/6-311++G(d,p) level. The vertical axis indicates the total energy (kJ mol^{-1}) of the isomers relative to that of isomer **a1**. The total energy was corrected with the zero-point vibrational energy.

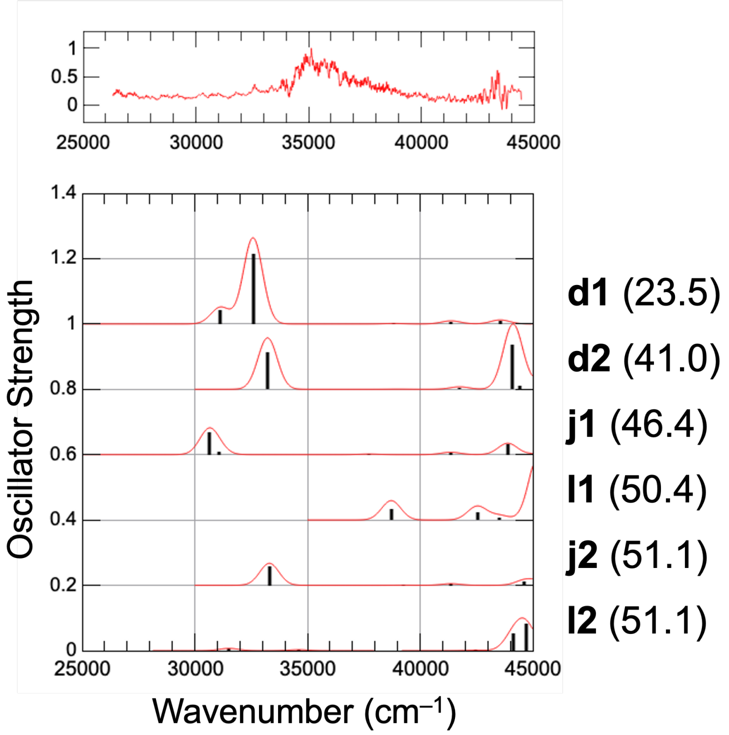

Figure S6. The electronic spectra calculated for the stable isomers of the *m/z* 171 ion for 1,3-DCB. The calculations were done at the M06-2X/6-311++G(d,p) level. The numbers in parentheses indicate the total energy (kJ mol^{-1}) of the isomers relative to that of isomer **a1**. The total energy was corrected with the zero-point vibrational energy.

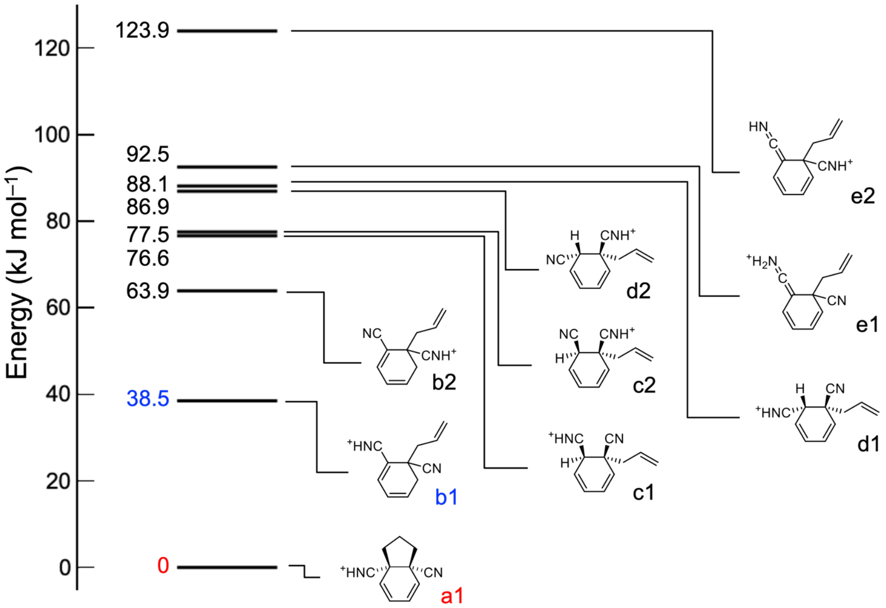

Figure S7. The structure and energy of the stable isomers of the *m/z* 171 ion with the allyl group at the *ipso* position of the cyano group for 1,2-DCB. The calculations were done at the M06-2X/6-311++G(d,p) level. The total energy was corrected with the zero-point vibrational energy.

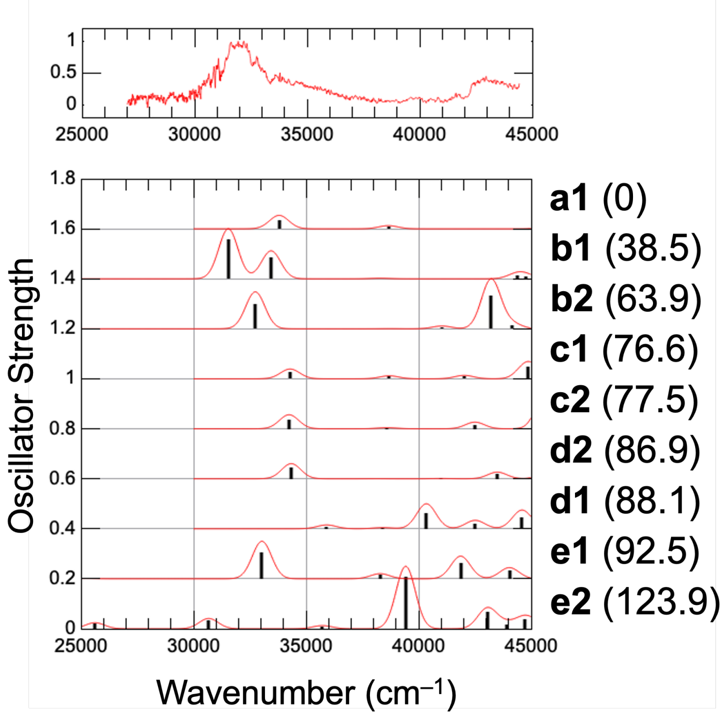

Figure S8. The electronic spectra calculated for the stable isomers of the *m/z* 171 ion for 1,2-DCB. The calculations were done at the M06-2X/6-311++G(d,p) level. The numbers in parentheses indicate the total energy (kJ mol^{-1}) of the isomers relative to that of isomer **a1**. The total energy was corrected with the zero-point vibrational energy.

Figure S9a displays the ^{1}H NMR spectra of 1,3-DCB and ATMS mixture in CD_3CN upon the irradiation of 0–5 h. From the spectra, we obtained the time profile of ATMS and 1,3-DCB (Figs. 9Sb and c). Different from the case of 1,2-DCB and 1,4-DCB, the NMR spectra of 1,3-DCB do not show clear signals assignable to the substitution product of the allyl group. On the other hand, the signals of ATMS and 1,3-DCB decreases with increasing the irradiation time (Figs. 9Sb and c), which indicates that ATMS and 1,3-DCB were converted to unidentified complex products.

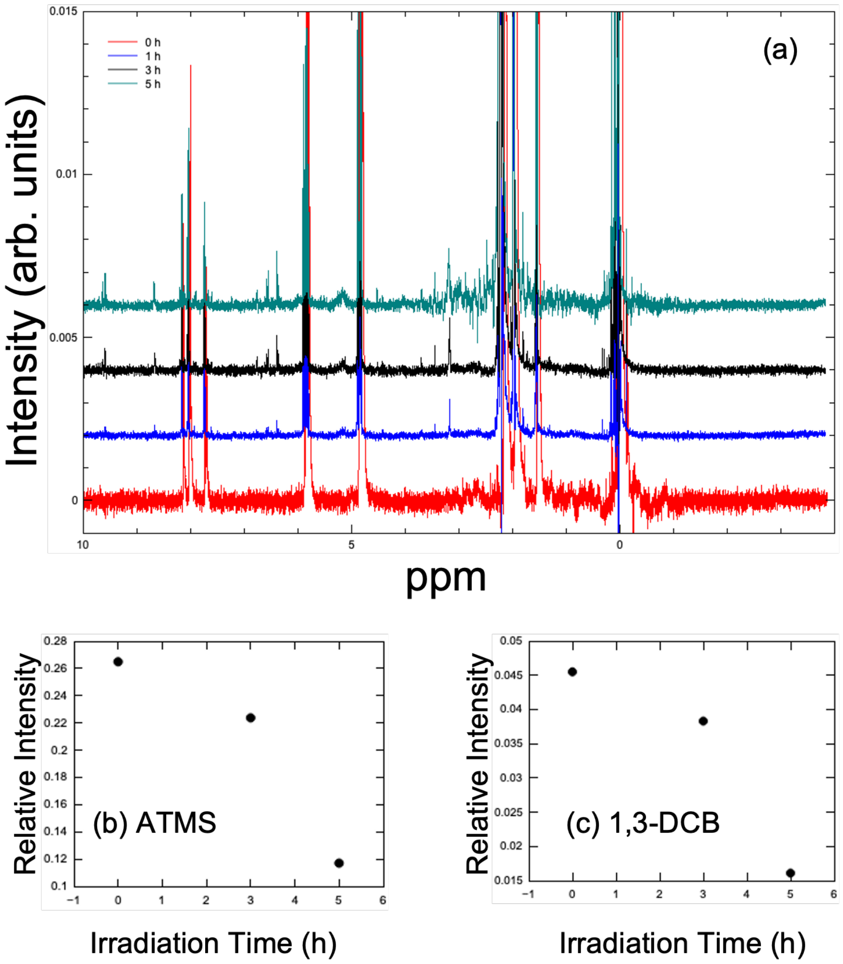

Figure S9. (a) The ^{1}H NMR spectra of 1,3-DCB and ATMS mixture in CD_3CN. (b, c) The time profile of the signal intensity of ATMS (5.74–5.88 ppm) and 1,3-DCB (7.65–7.74 ppm) relative to that of acetonitrile impurity (1.94 ppm) in deuterated acetonitrile.

Figure S10 shows the ^1H NMR spectra of 1,4-DCB and ATMS mixture in CD_3CN upon the irradiation of 0–5 h. The NMR spectra show clear signals assignable to the substitution product of the allyl group (4-allylbenzonitrile). Figure S11 displays the ^1H NMR spectra of 1,2-DCB and ATMS mixture in CD_3CN upon the irradiation of 0–5 h. Similarly to the case of 1,4-DCB, the irradiation of the reactant solution produced strong signals of the substitution product (2-allylbenzonitrile).

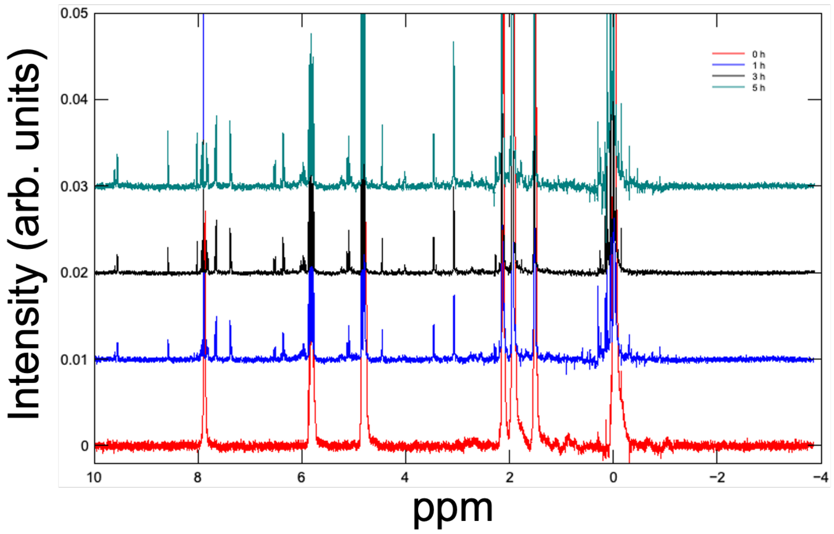

Figure S10. The ^1H NMR spectra of 1,4-DCB and ATMS mixture in CD_3CN.

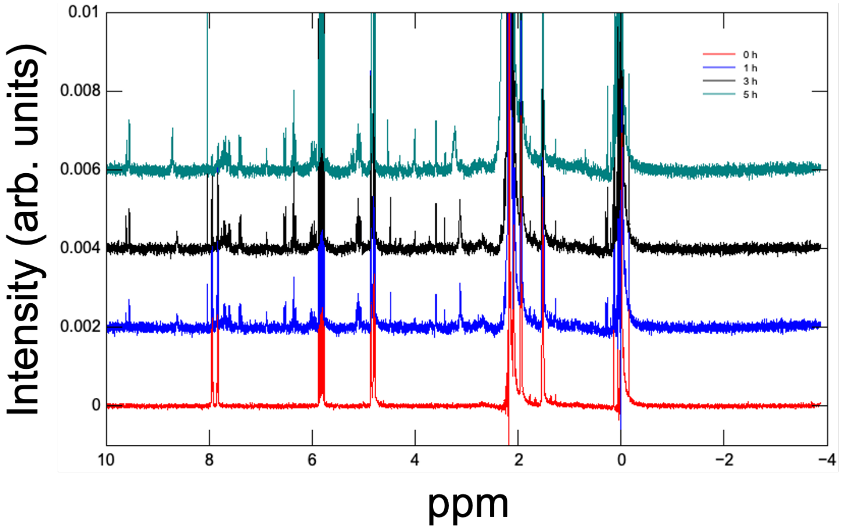

Figure S11. The ^1H NMR spectra of 1,2-DCB and ATMS mixture in CD_3CN.

Cartesian coordinates of isomer **a** for 1,4-DCB:

C	0.7120630	0.2926290	0.2964600
C	-0.2489320	0.7538520	1.4081130
C	-1.6880280	0.6724120	0.9963190
C	-2.0774250	-0.2819060	0.1371280
C	-1.1055690	-1.2436130	-0.4126230
C	0.2011600	-0.9951560	-0.3339160
H	-2.4061280	1.3528240	1.4377790
H	0.0084920	1.7628550	1.7322900
H	-1.4778590	-2.1463540	-0.8811130
H	0.9351460	-1.6771780	-0.7474410
C	-3.4588750	-0.4095450	-0.2311470
C	0.7464720	1.3270950	-0.7607680
C	2.1406940	0.1160410	0.8723040
H	2.0902110	-0.6878830	1.6108860
H	2.4059860	1.0392190	1.3967590
C	3.1742920	-0.1986170	-0.1696030
H	3.3737660	0.5787690	-0.9041370
C	3.8383880	-1.3476880	-0.2332420
H	4.5813610	-1.5281750	-1.0011360
H	3.6628070	-2.1416140	0.4864570
N	-4.5622440	-0.5272020	-0.5368040
N	0.7800930	2.1335120	-1.5798120
H	-0.1041740	0.0903450	2.2718090

Energy: -534.357810 hartree (zero-point energy corrected, M06-2X/6-311++G(d,p) with PCM (acetonitrile).

Cartesian coordinates of isomer **d** for 1,3-DCB:

C	-0.5806080	-2.2437640	-0.8916420
C	-1.7218530	-1.3804570	-0.6011310
H	-0.7558970	-3.1793150	-1.4076660
H	-2.6751840	-1.5838710	-1.0733130
C	0.9115990	-0.5035510	0.0496830
C	2.1277970	-0.5533440	0.8739760
C	1.1322360	0.5232950	-1.1064650
H	1.9360210	0.1395020	-1.7408310
H	0.2141290	0.5361310	-1.6989750
C	1.4615390	1.9045880	-0.6209020
H	2.4334720	2.0379190	-0.1506780
C	0.6342700	2.9386760	-0.7304620
H	-0.3422950	2.8314740	-1.1943020
H	0.9067310	3.9224900	-0.3668970
N	3.0818240	-0.5808900	1.5143330
C	-1.5802050	-0.3503230	0.2488020
C	-2.6869980	0.5014070	0.5566010
N	-3.5618650	1.2025590	0.8220370
C	-0.2684980	-0.1042870	0.9576740
H	-0.1708080	0.9421960	1.2487350
H	-0.2513740	-0.7085070	1.8717780
C	0.6614550	-1.8677550	-0.5719160
H	1.5210940	-2.4766010	-0.8277490

Energy: -534.359655 hartree (zero-point energy corrected, M06-2X/6-311++G(d,p) with PCM (acetonitrile).

Cartesian coordinates of isomer **b** for 1,2-DCB:

C	-2.6755570	0.0165700	0.4407320
C	-0.1940300	-0.4571190	-0.8440250
C	-1.2152400	-1.3257730	-0.9434610
C	-2.4341160	-1.1519920	-0.1631660
H	-3.6025730	0.1836690	0.9767550
H	-1.1351010	-2.1712170	-1.6166980
H	-3.1475180	-1.9651110	-0.1285720
C	0.9940390	-0.6372140	-1.6170210
C	-1.7117110	1.1631430	0.3262060
H	-1.9903440	1.7608880	-0.5507030
H	-1.7796860	1.8205440	1.1948150
C	-0.2526150	0.6770080	0.1828600
C	0.5687790	1.8125570	-0.2603170
N	1.2107140	2.7078860	-0.5870760
C	0.2921550	0.1719010	1.5520250
H	0.2101380	1.0025180	2.2600360
H	-0.3735330	-0.6271800	1.8868520
C	1.7071630	-0.3217790	1.4865460
H	2.4725340	0.4191970	1.2636900
C	2.0537030	-1.5902590	1.6756570
H	1.3102170	-2.3493330	1.8998230
H	3.0879290	-1.9082690	1.6160540
N	1.9539330	-0.7518810	-2.2441070

Energy: -534.358356 hartree (zero-point energy corrected, M06-2X/6-311++G(d,p) with PCM (acetonitrile).
